# Hemolysis after Oral Artemisinin Combination Therapy for Uncomplicated *Plasmodium falciparum* Malaria

**DOI:** 10.3201/eid2208.151905

**Published:** 2016-08

**Authors:** Florian Kurth, Tilman Lingscheid, Florian Steiner, Miriam S. Stegemann, Sabine Bélard, Nikolai Menner, Peter Pongratz, Johanna Kim, Horst von Bernuth, Beate Mayer, Georg Damm, Daniel Seehofer, Abdulgabar Salama, Norbert Suttorp, Thomas Zoller

**Affiliations:** Charité–Universitätsmedizin Berlin, Berlin, Germany (F. Kurth, T. Lingscheid, F. Steiner, M.S. Stegemann, S. Bélard, N. Menner, P. Pongratz, J. Kim, H. von Bernuth, B. Mayer, G. Damm, D. Seehofer, A. Salama, N. Suttorp, T. Zoller);; Swiss Tropical and Public Health Institute, Basel, Switzerland (T. Zoller)

**Keywords:** malaria, uncomplicated malaria, Plasmodium falciparum, parasites, hemolysis, artemisinin, oral artemisinin combination therapy, clinical study, hemoglobin, haptoglobin, lactate dehydrogenase, anemia

## Abstract

Episodes of delayed hemolysis 2–6 weeks after treatment of severe malaria with intravenous artesunate have been described. We performed a prospective observational study of patients with uncomplicated malaria to investigate whether posttreatment hemolysis also occurs after oral artemisinin-based combination therapy. Eight of 20 patients with uncomplicated malaria who were given oral artemisinin-based combination therapy met the definition of posttreatment hemolysis (low haptoglobin level and increased lactate dehydrogenase level on day 14). Five patients had hemolysis persisting for 1 month. Patients with posttreatment hemolysis had a median decrease in hemoglobin level of 1.3 g/dL (interquartile range 0.3–2.0 g/dL) in the posttreatment period, and patients without posttreatment hemolysis had a median increase of 0.3 g/dL (IQR −0.1 to 0.7 g/dL; p = 0.002). These findings indicate a need for increased vigilance for hemolytic events in malaria patients, particularly those with predisposing factors for anemia.

## Abstract 2

Clinicians should be aware of hemolysis in malaria patients with anemia who were given these drugs.

Artemisinin-based drugs are the mainstay of current antimalarial treatment and play a key role in the World Health Organization (WHO) global strategy to reduce malaria illness and death caused by malaria. These drugs act rapidly against *Plasmodium* spp. and are usually well tolerated. Artemisinin-based combination therapies (ACTs) are the recommended first-line treatment for uncomplicated malaria in most countries (*1*).

Episodes of delayed hemolysis 2–6 weeks after treatment for severe malaria with intravenous artesunate have been observed in non–malaria-immune patients in Europe (*2*). This phenomenon, recently referred to as postartemisinin-delayed hemolysis (PADH) (*3*,*4*), has been confirmed in other nonimmune patients (*4*,*5*) and in children in Africa (*6*). Approximately 20%–30% of nonimmune patients given intravenous artesunate show signs of PADH that vary in intensity and duration (*5*,*7*). Hemolysis is usually self-limiting, but patients need to be actively followed up because transfusion of erythrocytes and rehospitalization might be necessary (*2*,*5*).

The pathophysiology of hemolysis after artemisinin therapy is not fully understood. Once-infected erythrocytes that have been cleared of parasites in the spleen have a shorter life span and play a role. Patients with higher concentrations of once-infected erythrocytes after artemisinin treatment are at higher risk for PADH (*4*). However, other features of posttreatment hemolysis, such as prolonged hemolytic reactions over several weeks (*2*), are not explained by this mechanism. Several reports suggest involvement of a drug-dependent autoimmune hemolysis mechanism (*8*), but systematic investigations have not been performed in most published cases (*2*,*9*). Given the key role of artemisinins in malaria treatment, WHO calls for prospective clinical studies and further research to improve the understanding of delayed hemolysis after artemisinin therapy (*10*).

Only 2 single cases and 2 patients in a recent analysis of surveillance data in the United States have been reported with signs of delayed hemolysis after oral ACT treatment (*11*–*13*). We hypothesize that delayed hemolysis occurs not only after intravenous treatment for severe malaria but also in a substantial number of patients given oral ACTs for uncomplicated malaria. Because delayed hemolysis has not been captured by safety studies on ACTs, we assume that delayed hemolysis after oral ACTs is less pronounced and occurs to a subclinical degree.

We conducted a study of patients with uncomplicated *Plasmodium falciparum* malaria to investigate the clinical, laboratory, and immunohematologic features of hemolysis and anemia during and after antimalarial treatment. This article presents data for patients investigated during the first 12 months of this ongoing study.

## Methods

This prospective observational study was conducted at the University Hospital of Charité–Universitätsmedizin Berlin (Berlin, Germany). The study protocol was approved by the ethical committee of Charité–Universitätsmedizin Berlin and is registered at the WHO International Clinical Trials Registry Platform (DRKS00007104). All laboratory analyses and parasitologic examinations were performed in accredited laboratories at Charité-Universitätsmedizin, Berlin.

All patients who sought treatment at the hospital and were found to have microscopically confirmed uncomplicated *P. falciparum* malaria were included in the study after written informed consent was obtained. Patients were excluded if they had received antimalarial treatment (excluding prophylaxis) within 12 weeks before inclusion; had medical conditions that potentially cause hemolysis (e.g., glucose-6-phosphate dehydrogenase deficiency, hemoglobinopathy, mechanical heart valve, lymphoproliferative disease); or were taking medication that potentially causes hemolysis.

Patients were seen for study visits at admission before treatment (day 0). After the first treatment, they were seen again after the last treatment dose on day 3, on day 7 (range day 6–day 10), and on day 14 (range day 14–day 20). If symptoms or signs of hemolysis were detected, patients were seen on day 30 (range day 27–day 31) and thereafter if clinically indicated.

Study visits included obtaining a medical history and conducting a physical examination. Laboratory investigations were parasitologic (thick and thin blood smears), hematologic (differential blood count), and biochemical (haptoglobin, lactate dehydrogenase [LDH], C-reactive protein, potassium, and sodium levels and renal and liver function tests) examinations; screening for glucose-6-phosphate dehydrogenase deficiency; and immunohematologic examinations (direct and indirect antiglobulin test, including testing with enzyme-treated erythrocytes).

This analysis evaluated data for all patients given oral ACTs during the first 12 months of the study. The primary objective was to assess the proportion of patients with posttreatment hemolysis, which was defined as a low haptoglobin level (<0.3 g/L) and an LDH level above the age-dependent upper normal level 14 days after treatment. Secondary objectives were to compare in patients with posttreatment hemolysis and those without it possible risk factors (age, ethnicity, sex, initial parasitemia) and the course of anemia (hemoglobin [Hb] level, reticulocyte production index) during treatment, after treatment, and overall. Hemolysis with a loss of Hb >1.5 g/dL during days 3–14 was classified as uncompensated hemolysis; hemolysis without a decrease in Hb level or a decrease <1.5 g/dL during days 3–14 was classified as compensated hemolysis.

Differences between patients with and without signs of posttreatment hemolysis and between patients with compensated and uncompensated hemolysis were analyzed by using the Mann-Whitney U test for continuous data and the Fisher exact test for binary data at a 2-sided significance level of α = 0.05. Data are presented as median and interquartile range (IQR). Statistical analysis was performed by using JMP version 7.0 (SAS Institute Inc., Cary, NC, USA).

The sample size of the ongoing study was calculated to detect an incidence of posttreatment hemolysis of 20% with a 95% CI, ± 7.5% precision, and 15% lost to follow-up. This calculation resulted in a sample size of 130 patients. This study evaluated 27 patients, which represented 21% of the intended total sample size. Because we could find no published prospective data for hemolysis after oral ACT treatment, we decided to communicate the findings of this interim analysis before completion of the study.

## Results

During May 2014–April 2015, a total of 27 patients with uncomplicated *P. falciparum* malaria and a standard 3-day treatment course of oral ACT were included in the study. All malaria infections had been acquired in Africa, and none of the patients had taken antimalarial prophylaxis. Six patients did not complete all necessary follow-up visits, and 1 patient was excluded because of sickle cell disease. Twenty patients with >4 study visits until day 20 were available for this interim analysis; of these patients, 3 were children (Table). All patients showed rapid clinical improvement with clearance of peripheral asexual parasitemia no later than 72 hours after initiation of treatment. There were no treatment failures.

**Table:** Baseline characteristics and follow-up laboratory data for patients with uncomplicated *Plasmodium falciparum* malaria who were given ACT*

Characteristic	All, n = 20	Without posttreatment hemolysis, n = 12	With posttreatment hemolysis, n = 8	p value	With compensated posttreatment hemolysis, n = 4	With uncompensated posttreatment hemolysis, n = 4	p value
Baseline							
Age, y	35 (26–40)	31 (17–40)	38 (30–43)	0.18	32 (22–42)	40 (27–46)	0.15
Children	3/20 (15.0)	3/12 (25.0)	0/8 (0)	0.24	0/4 (0)	0 (0)	
African ethnicity	13/20 (65.0)	9/12 (75.0)	4/8 (50.0)	0.35	4/4 (100.0)	0/4 (0)	0.001
Female sex	9/20 (45.0)	7/12 (58.3)	2/8 (25.0)	0.19	2/4 (50.0)	0/4 (0)	0.42
Treatment with ARM/LUM	5/20 (25.0)	4/12 (33.3)	1/8 (12.5)	0.60	0/4 (0)	1/4 (25.0)	1.0
Treatment with DHA/PPQ	15/20 (75.0)	8/12 (75.0)	7/8 (87.5)	0.60	4/4 (100.0)	3/4 (75.0)	1.0
Parasitemia†	0.4 (0.2–1.1)	0.3 (0.1–0.9)	0.9 (0.4–1.4)	0.12	1.15 (0.4–1.9)	0.8 (0.2–1.1)	0.40
Hb d0‡	12.5 (11.1–14.0)	11.3 (10.5–13.5)	13.1 (12.5–14.1)	0.11	12.7 (12.4–13.8)	13.7 (12.6–14.6)	0.30
Laboratory follow-up‡							
Hb d3	12.2 (10.6–13.6)	11.1 (9.7–12.7)	13.2 (12.2–14.3)	0.02	12.8 (11.9–13.4)	14.2 (12.5–14.6)	0.15
Hb d7	12.1 (11.1–13.0)	11.7 (10.5–12.5)	12.5 (11.9–12.6)	0.33	12.6 (11.8–13.1)	12.7 (11.5–12.9)	0.66
Hb d14	12.0 (10.9–12.6)	11.7 (10.5–12.6)	12.2 (11.9–12.6)	0.33	12.5 (12.2–12.8)	11.9 (10.5–12.5)	0.11
ΔHb d0–d3	−0.4 (−0.8 to 0.0)	−0.5 (−1.0 to −0.3)	−0.1 (−0.6 to 0.6)	0.07	−0.4 (−1.1 to 0.7)	0.1 (−0.2 to 0.6)	0.40
ΔHb d3–d7	0.0 (−0.8 to 0.5)	0.3 (0.3–0.8)	−0.8 (−1.5 to −0.1)	0.007	−0.3 (−0.6 to 0.2)	−1.5 (−1.7 to −1.0)	NA
ΔHb d7–d14	0.1 (−0.5 to 0.5)	0.3 (0.1–0.5)	−0.4 (−0.9 to 0.1)	0.04	0.0 (−0.4 to 0.4)	−0.8 (−1.2 to −0.3)	NA
ΔHb d3–d14	0.0 (−0.7 to 0.5)	0.3 (−0.1 to 0.7)	−1.3 (−2.0 to −0.3)	0.002	−0.3 (−0.6 to 0.3)	−1.9 (−2.6 to −1.9)	NA
ΔHb d0–d14	−0.7 (−1.1 to 0.1)	−0.4 (−0.8 to 0.4)	−1.3 (−2.1 to −0.3)	0.03	−0.4 (−1.3 to 0.2)	−1.9 (−2.8 to −1.3)	NA
LDH d7, U/L	250 (225–331)	244 (222–274)	327 (229–407)	0.16	329 (211–495)	327 (248–381)	0.77
LDH d14, U/L	256 (210–283)	210 (197–247)	280 (256–365)	0.006	273 (255–394)	298 (234–365)	1.0
RPI d3	0.5 (0.3–0.7)	0.4 (0.2–0.7)	0.7 (0.4–0.7)	0.22	0.7 (0.4–0.7)	0.6 (0.5–0.7)	1.0
RPI d7	1.4 (0.9–1.6)	1.4 (0.9–1.5)	1.3 (0.8–2.0)	0.66	1.7 (1.2–2.7)	1.0 (0.4–1.8)	0.24
RPI d14	1.4 (1.0–1.8)	1.1 (1.0–1.6)	1.9 (1.4–2.6)	0.015	2.5 (1.9–2.9)	1.5 (1.1–1.9)	0.04

*Values are median (interquartile range) or n/N (%). ACT, artemisinin-based combination therapy; ARM, artemether; d, day; Δ, period between indicated days; DHA, dihydroartemisinin; Hb, hemoglobin; LDH, lactate dehydrogenase; LUM, lumefantrine; NA, not applicable; PPQ, piperaquine; RPI, reticulocyte production index. †Percentage of erythrocytes infected. ‡Hb levels are in grams/deciliter.

The criteria for posttreatment hemolysis (haptoglobin and LDH levels) on day 14 were met by 8 (40%) of 20 patients. An additional 2 patients showed signs of in vitro hemolysis on day 14 (increased LDH and potassium levels but haptoglobins level within reference ranges). The LDH values of these patients were excluded from further analysis and these patients were classified as patients without hemolysis. Patient characteristics showed no differences between those with and without hemolysis, with the exception of slightly higher Hb levels on day 0 and day 3 in patients with posttreatment hemolysis (Table).

**Figure 1:**
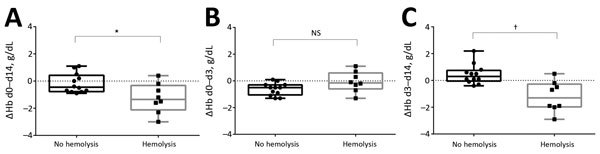
Changes in hemoglobin levels (ΔHb) for patients with and without posttreatment hemolysis after treatment with oral artemisinin-based combination therapy for uncomplicated *Plasmodium falciparum* malaria. A) Day (d) 0 to d 14 (overall); B) d 0 to d 3 (treatment period); C) d 3 to d 14 (posttreatment period). Horizontal lines indicate median values, boxes indicate interquartile ranges, whiskers indicate ranges, and solid squares and circles indicate individual patient data points. The Mann-Whitney U test was used for comparative analysis. *p<0.05; †p<0.01; NS, not significant.

After treatment (during days 3–14), patients with posttreatment hemolysis showed a decrease in Hb level (median change −1.3 g/dL, IQR −2.0 to −0.3), and patients without posttreatment hemolysis showed an increase in Hb level (median change 0.3 g/dL, IQR −0.1 to 0.7; p = 0.002) (Figure 1, panel C). During treatment (during days 0–3), patients with posttreatment hemolysis showed a tendency toward a smaller decrease in Hb level (median change −0.15 g/dL, IQR −0.6 to 0.6) than did patients without posttreatment hemolysis (median change −0.5 g/dL, IQR −1.0 to −0.3; p = 0.07) (Figure 1, panel B). Overall (during days 0–14), patients with posttreatment hemolysis showed a larger decrease in Hb level (median change −1.35 g/dL, IQR −2.1 to −0.3) than did patients without posttreatment hemolysis (median change −0.45 g/dL, IQR −0.8 to 0.4; p = 0.03) (Figure 1, panel A).

**Figure 2:**
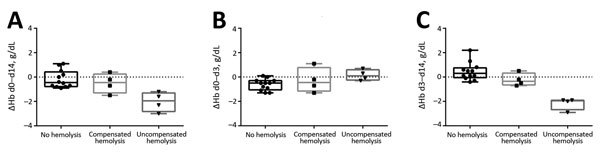
Changes in hemoglobin levels (ΔHb) for patients without posttreatment hemolysis, with compensated posttreatment hemolysis, and with uncompensated posttreatment hemolysis after treatment with oral artemisinin-based combination therapy for uncomplicated *Plasmodium falciparum* malaria. A) day (d) 0 to d 14 (overall); B) d 0 to d 3 (treatment period); C) d 3 to d 14 (posttreatment period). Horizontal lines indicate median values, boxes indicate interquartile ranges, whiskers indicate ranges, and solid squares, circles, and triangles indicate individual patient data points.

Analysis of the course of anemia in the 8 patients with posttreatment hemolysis showed that a decrease in Hb level during days 3–14 occurred in only 4 patients (uncompensated hemolysis). The other 4 patients with hemolysis maintained stable Hb levels during days 3–14 (compensated hemolysis) (Figure 2, Panel C). Consistent with this observation, we found that patients with compensated posttreatment hemolysis showed a higher reticulocyte production index on day 14 than did patients with uncompensated posttreatment hemolysis or without hemolysis on day 14. No differences were observed in median LDH levels and initial parasitemia between patients with compensated or uncompensated posttreatment hemolysis (Table). All patients with compensated posttreatment hemolysis were of African ethnicity, and all patients with uncompensated posttreatment hemolysis were Caucasian.

**Figure 3:**
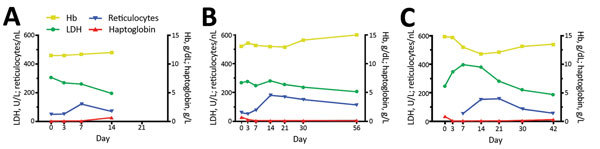
Laboratory values over time for exemplary patients with and without posttreatment hemolysis after treatment with oral artemisinin-based combination therapy for uncomplicated *Plasmodium falciparum* malaria. A) Patient without posttreatment hemolysis, B) patient with compensated posttreatment hemolysis, and C) patient with uncompensated posttreatment hemolysis. Hb, hemoglobin; LDH, lactate dehydrogenase.

Five patients with posttreatment hemolysis (4 patients with uncompensated hemolysis and 1 patient with compensated hemolysis) were followed-up until day 30. All of these patients had persistent low haptoglobin levels (<0.3 g/L) on day 30, and 2 patients still had LDH levels above the age-dependent upper reference level on day 30. Exemplary cases of a patient without hemolysis (Figure 3, panel A), a patient with compensated hemolysis (Figure 3, panel B), and a patient with uncompensated hemolysis (Figure 3, panel C) show the course of laboratory values over time. The patient with compensated hemolysis had a low haptoglobin level (<0.3 g/L) and an increased reticulocyte count 8 weeks after treatment on day 56 (Figure 2, panel B).

Immunohematologic testing showed that serum samples from 5 (25%) of 20 patients were reactive only with enzyme-treated erythrocytes after therapy (3 patients with and 2 patients without posttreatment hemolysis). The direct antiglobulin test result was weakly positive for 3 patients; none of them had posttreatment hemolysis. None of the patients with hemolysis showed coating of erythrocytes with IgG, IgM, or C3d.

## Discussion

Hemolytic anemia after treatment of severe malaria with intravenous artesunate has been described in malaria-endemic and non–malaria-endemic countries. However, evidence of hemolytic anemia after treatment of malaria with oral ACTs is limited to 2 case reports. Data from the current prospective study confirm our hypothesis that delayed posttreatment hemolysis also occurs after oral artemisinin treatment and provide insight into its frequency and clinical course. In 40% of the patients in our study with uncomplicated malaria and oral ACT treatment, laboratory signs of hemolysis were detected 2 weeks after therapy. In 5 patients, hemolysis persisted 1 month after treatment. Patients with posttreatment hemolysis showed a larger decrease in Hb levels after treatment than did patients without hemolysis. The intensity of hemolysis was mild compared with that after intravenous artesunate. In many reported cases of PADH after intravenous artesunate, patients received blood transfusions (*2*,*9*). In other studies, patients with hemolysis after oral ACT treatment had decreases in Hb levels of 2.1 g/dL–3.6 g/dL in the posttreatment period (*11*–*13*).

The decrease in Hb levels during treatment in the current study was smaller in patients with posttreatment hemolysis than in patients without posttreatment hemolysis. Consistent with this finding, we found that the patient group with the largest decrease in Hb levels after treatment (i.e., patients with uncompensated hemolysis) showed a small increase in Hb levels during treatment (Figure 2). This observation could be explained by involvement of once-infected erythrocytes: during treatment, erythrocytes are spared by removal of parasites without destruction of the cell. The Hb level therefore remains stable. After treatment, the once-infected, pitted erythrocytes hemolyze because of their shorter life spans, which results in a postponed loss of Hb during the posttreatment period (*4*). Our data therefore give additional support to the relevance of this mechanism as a cause of late hemolysis.

Half of the patients with posttreatment hemolysis showed erythropoietic activity at day 14 that was sufficient for compensating the postponed loss of Hb. These patients were all of African origin, unlike those with uncompensated hemolysis, who were all Caucasian. The reason for this observation is unknown. Malaria-related dyserythropoiesis (*14*) might be less pronounced in African patients than in European patients. Impairment of erythropoiesis by artemisinins has been described in vitro (*15*), but no differences regarding ethnicity have been reported.

Different reported clinical courses of delayed hemolysis after artemisinin therapy suggest involvement of mechanisms other than pitting (*4*,*16*). In some patients, the decrease in Hb level far exceeds the loss of erythrocytes expected from destruction of once-infected erythrocytes (*16*). In a recent case report, drug-dependent autoimmune hemolysis was reported as a probable cause of PADH (*8*). Several other reports failed to demonstrate immune-mediated hemolysis or drug-induced antibodies in patients with severe malaria (*2*,*9*,*17*). In our patients, results from immunohematologic testing were inconclusive. No antibody or complement coating of erythrocytes was found that could trigger bystander hemolysis of uninfected erythrocytes.

Baseline Hb levels were comparatively high in our patients. The mild loss of Hb therefore did not result in clinical symptoms. However, in settings in which chronic anemia is common because of concomitant infections and nutritional deficiencies, posttreatment hemolysis after antimalarial treatment might be a clinically relevant factor. Recently, a large study in Nigeria reported a >5% decrease in hematocrit levels in 23% of African children with uncomplicated malaria 14–28 days after ACT treatment (*18*). Although no further assessments were performed in this study, the authors assumed an association with postartemisinin hemolysis. Further prospective investigations of this phenomenon in malaria-endemic areas are needed and should include markers for detection of hemolysis.

The dataset used for this analysis has several limitations. The most relevant limitation is that this study has, so far, not included patients who are receiving oral antimalarial drugs other than ACTs. At this time, we cannot rule out that similar hemolytic reactions occur after non-ACT antimalarial treatment because no prospective studies are available with a comparable method to detect hemolysis. More data on posttreatment anemia, hemolysis, and erythropoiesis after non-ACT treatment are therefore needed for comparison. However, this analysis also included patients with no evidence of posttreatment hemolysis after malaria and ACT therapy; it is therefore unlikely that hemolysis generally occurs after antimalarial therapy. The main objective of this study was to prospectively collect evidence for posttreatment hemolysis after oral ACT treatment. Some uncertainty might arise from the limited number of patients regarding other conclusions, such as different reticulocyte responses in patients from Africa and Europe. These conclusions have to be confirmed with larger sample sizes.

In conclusion, our study provides evidence that a mild form of posttreatment hemolysis commonly occurs after oral ACT treatment for uncomplicated malaria. The role of this observation for clinical practice in malaria-endemic and non–malaria-endemic settings remains to be defined but should prompt increased vigilance for hemolytic events, particularly for patients with preexisting anemia or those for whom mild anemia constitutes a clinical problem. Larger studies are needed to investigate observations and hypotheses concerning underlying pathophysiology and to eventually identify potential risk factors.
